# Glacial vicariance in Eurasia: mitochondrial DNA evidence from Scots pine for a complex heritage involving genetically distinct refugia at mid-northern latitudes and in Asia Minor

**DOI:** 10.1186/1471-2148-7-233

**Published:** 2007-11-22

**Authors:** Krassimir Naydenov, Sauphie Senneville, Jean Beaulieu, Francine Tremblay, Jean Bousquet

**Affiliations:** 1 Chaire industrielle CRSNG-UQAT-UQAM, Université du Québec en Abitibi-Témiscamingue, 445 Bd. de l'Université, Rouyn-Noranda (Québec), J9X 5E4, Canada; 2 Chaire de recherche du Canada en génomique forestière et environnementale and Centre d'étude de la forêt, Pavillon Charles-Eugène-Marchand, Université Laval, Québec (Québec), G1K 7P4, Canada; 3 Natural Resources Canada, Canadian Forest Service, Canadian Wood Fibre Centre, 1055 du PEPS, PO Box 10380, Stn. Sainte-Foy, Quebec (Quebec), G1V 4C7, Canada

## Abstract

**Background:**

At the last glacial maximum, Fennoscandia was covered by an ice sheet while the tundra occupied most of the rest of northern Eurasia. More or less disjunct refugial populations of plants were dispersed in southern Europe, often trapped between mountain ranges and seas. Genetic and paleobotanical evidences indicate that these populations have contributed much to Holocene recolonization of more northern latitudes. Less supportive evidence has been found for the existence of glacial populations located closer to the ice margin. Scots pine (*Pinus sylvestris *L.) is a nordic conifer with a wide natural range covering much of Eurasia. Fractures in its extant genetic structure might be indicative of glacial vicariance and how different refugia contributed to the current distribution at the continental level. The population structure of Scots pine was investigated on much of its Eurasian natural range using maternally inherited mitochondrial DNA polymorphisms.

**Results:**

A novel polymorphic region of the Scots pine mitochondrial genome has been identified, the intron 1 of *nad7*, with three variants caused by insertions-deletions. From 986 trees distributed among 54 populations, four distinct multi-locus mitochondrial haplotypes (mitotypes) were detected based on the three *nad7 *intron 1 haplotypes and two previously reported size variants for *nad1 *intron B/C. Population differentiation was high (*G*_ST _= 0.657) and the distribution of the mitotypes was geographically highly structured, suggesting at least four genetically distinct ancestral lineages. A cosmopolitan lineage was widely distributed in much of Europe throughout eastern Asia. A previously reported lineage limited to the Iberian Peninsula was confirmed. A new geographically restricted lineage was found confined to Asia Minor. A new lineage was restricted to more northern latitudes in northeastern Europe and the Baltic region.

**Conclusion:**

The contribution of the various ancestral lineages to the current distribution of Scots pine was asymmetric and extant endemism reflected the presence of large geographic barriers to migration. The results suggest a complex biogeographical history with glacial refugia shared with temperate plant species in southern European Peninsulas and Asia Minor, and a genetically distinct glacial population located more North. These results confirm recent observations for cold tolerant species about the possible existence of refugial populations at mid-northern latitudes contributing significantly to the recolonization of northern Europe. Thus, Eurasian populations of nordic plant species might not be as genetically homogenous as assumed by simply considering them as offsets of glacial populations located in southern peninsulas. As such, they might have evolved distinctive genetic adaptations during glacial vicariance, worth evaluating and considering for conservation.

## Background

The cold periods of the Pleistocene have had a dramatic impact on most organisms in temperate regions. Whereas the Artic ice sheet began to grow about 2.5 Myr ago, more severe climatic oscillations occurred mainly during the last 700 000 year [1] with the consequence that the European biota has experienced repeatedly some drastic climate changes. Tree species responded through migration to regions where environmental conditions allowed them to survive [2,3]. Phylogeography investigates on the spatio-temporal dynamics of populations. It relies on inference from two main sources of information, i) macrofossils and pollen in sediment profiles, ii) population structure in DNA markers ideally inferred at the sequence level. Cytoplasmic markers, which are uniparently inherited in most plant species, are especially well suited for phylogeographic studies. They helped infer the number of genetically distinct ancestral lineages, their location during the last glacial maximum, and the postglacial migration routes for several tree and plant species in Europe [e.g. [4-11]]. These studies and others based on fossil evidence suggested that most plant and tree refuges were located in southern European peninsulas and the Balkans [12-15]. Glacial refugia and recolonization routes were found to coincide often for a number of plant and animal species [[3,16] and [17]]. In North America, common patterns are also starting to emerge and much knowledge has been rapidly gained from the analysis of species with wide distributions [e.g. [18-20]]. However, there is now increasing evidence that some European species were established much further north and east than previously assumed during glacial time [21-25]. Similar inferences have also been made in North America, where genetic and palynological evidence is accumulating for the presence of glacial populations much closer to the ice fronts as once thought [e.g. [26-29]]. Phylogeographic studies in Europe also indicate asymmetric contributions of glacial refugia to Holocene colonization: some ancestral lineages remained endemic, because of geographic barriers limiting migration, while others contributed considerably to the postglacial colonization of more northern latitudes [[3,6,8] and [30]].

Scots pine (*Pinus sylvestris *L., subgenus *Diploxylon*) is a long-lived evergreen monoecious forest tree species growing in a large variety of ecological conditions from western Europe to Asia. Of all pine species, it has the largest distribution [31,32]. Its natural range extends northwards from Spain to northern Fennoscandia, and westwards from longitudes 5°W in Spain and Scotland to 135°E in Siberia. While most of its natural range is contiguous, there are also large populations of Scots pine disconnected from the main natural range such as in Scotland, Spain, France, Turkey, and former Yugoslavia. Extensive variations in phenotypic traits as well as geographic races and types have been reported [33-37]. Much of this variation has been usually attributed to adaptation to edaphic and climatic factors [35,38]. Studies carried out over the last 30 years using isozymes, terpenes and flavonoid markers have also revealed the existence of distinct evolutionary units in Scots pine, which might be indicative of isolation in different glacial refugia. Although these studies were not entirely congruent and not aimed at deciphering effects related to glacial and postglacial history, they helped define biogeographical hypotheses for the western and northwestern European range of Scots pine [39-45]. Because of the regional scope of many of these studies and the different phenotypic or genetic attributes used, much biogeographical knowledge on the glacial and postglacial history of Eurasia remains to be gained from analysing the broad geographic distribution of Scots pine and by using homologous gene markers with stronger historical imprints.

In the Pinaceae, mtDNA is maternally inherited and dispersed through seeds only [46,47]. This mode of dispersion helps avoid the problems with inferring history from present-day distributions of nuclear or cpDNA markers because the latter are dispersed by both seeds and pollen in the Pinaceae, which produce a more rapid breakdown of the historical genetic signatures induced by glacial vicariance. However, one of the major difficulties one faces with plant mtDNA is the low level of variation in their exons and introns [48,49], which limits the detection of variation between and within species at the sequence level [e.g. [47,50]]. An indel has been discovered in the intron B/C of the mitochondrial gene *nad1 *in Scots pine, which allowed identifying a genetically distinct ancestral lineage in the Iberian Peninsula [6]. Restriction fragment length polymorphisms (RFLP) of a region encompassing the mitochondrial gene *cox1 *have also been found in populations of Scots pine from western Europe [5,51], which helped identifying two genetically distinct groups of populations each distributed on a large latitudinal range. The possibility for a refuge at northern latitudes was suggested in a previous study [5]. Given the little diversity observed in the mtDNA regions surveyed and the population sampling limited mostly to western Europe, it is likely that the pan-Eurasian Scots pine possesses more genetically distinct ancestral lineages. Such evidence would contribute towards a more complete understanding of the historical factors and processes leading to the current distribution of species genetic diversity throughout Eurasia.

In an effort to discern from a phylogeographic perspective the possible existence and contribution of mid-northern refugia to the glacial and postglacial history of Eurasian biota, we investigated the extant genetic structure of Scots pine with a set of populations covering much of the natural range in Europe and also in Asia. We also report on a novel polymorphic mtDNA region in Scots pine, the intron 1 of the gene *nad7*, which is indicative of unknown genetically distinct maternal lineages. The location of one of these lineages in northern Europe supports the view that Quaternary refugia located outside European peninsulas at mid-northern latitudes might have played a significant role in the Holocene recolonization of Europe [22].

## Results

### mtDNA polymorphisms and population differentiation

Out of 15 mtDNA regions surveyed (Table 1), intraspecific polymorphism was detected for only two loci in Scots pine. Polymorphism was detected *de novo *for the intron 1 of *nad7 *with three length variants. They were 1175, 1170 and 1143 bp long and are hereafter called haplotypes A, B, and C, respectively. The size differences were caused by two single indels: one of 32 bp exclusive to haplotype C and one of 5 bp exclusive to haplotype B (Table 2). As previously reported from a more limited sampling [6], two size variants were detected for the intron B/C of *nad1 *among the 986 samples analysed herein. These fragments differed by a 31 bp indel and are hereafter called haplotypes A (217 bp) and B (248 bp). A third variant restricted to Italy was recently reported for this gene [30]. It was not detected in our sampled trees which were all from outside the Italian Peninsula. Primers for these two mtDNA regions were also tested on 100 to 500 individuals for each of five other pines of the subgenus *Diploxylon *(*P. resinosa, P. mugo, P nigra, P. heldreichii*, and *P. contorta*) and positive amplifications were obtained (data not shown). The first four species belong to the section *Pinus*, but the first three are members of subsection *Pinus *(as for Scots pine) and *Pinus heldreichii *is member of subsection *Pinaster*. As for *P. contorta*, it belongs to section *Trifoliae*, subsection *Contortae*. Fragment length polymorphism was detected for both *nad1 *intron B/C and *nad7 *intron 1 in *P. heldreichii*. For *P. nigra*, only *nad1 *intron B/C showed polymorphism. No polymorphism was detected in *P. mugo *and *P. resinosa*. For *P. contorta*, polymorphism was only observed for *nad7 *intron 1 involving the same minisatellite-like marker as previously reported for this species and the closely related *P. banksiana *[20].

**Table 1:** Mitochondrial regions tested^1^.

Genomic region	Annealing temperature (°C)	PCR product size (bp)	Restriction enzymes tested	Primer source
*matR *(intron1)	58	550	*Sau*3AI, *Rsa*I, *Hind*III, *Mse*I, *Taq*I, *Bst*UI	[47]
*mh05*	59	1500 ^4^	-	[91]
*mh09*	62	180	-	[91]
*mh09'*	62	900 ^4^	-	[91]
*mh33*	55	900 ^4^	-	[91]
*mh35*	59	Not amplified	-	[91]
*mh44*	59	Not amplified	-	[91]
*nad1 *intron B/C, primers H/I ^2^	55	217–248	-	[6]
*nad3 *intron 1	58	120	-	[92]
*nad3*-*rps12 *(i.r.) ^3^	58	360	-	[92]
*nad5 *intron 1	55	1100	*Sau*3AI, *Rsa*I, *Hind*III, *Mse*I, *Taq*I, *Bst*UI,	[47]
*nad5 *intron 4	59	900	*Sau*3AI, *Rsa*I, *Hind*III, *Mse*I, *Taq*I, *Bst*UI, *Tru9*I	[93]
*nad7 *intron 1	61	1200	*Sau*3AI, *Rsa*I, *Hind*III, *Taq*I, *Bst*UI, *Tru9*I	[47]
*SSU rRNA *region V1	62	400	-	[94]
*SSU rRNA *region V7	50	250	-	[94]

^1^Targeted regions, annealing temperatures, expected size of PCR products and restriction enzymes tested for primer pairs used to amplify 15 mtDNA regions of Scots pine.

^2^*nad1 *intronB/C have been amplified (following Demesure *et al *[95] protocol) and tested with different restriction enzymes, only internal primers H and I of Soranzo *et al *[6] have shown polymorphism.

^3^Intergenic region.

^4^Multiple fragments.

**Table 2:** Mitochondrial DNA sequence polymorphisms detected in Scots pine^1^.

Mitotype	*nad7 *intron 1 (positions 621–681)	*nad1 *intron B/C (positions 101–137)
AA	GGGATGCGTAAGCAGGCTCGACTGTTAAGGAGAGGGGCAAATAAGTAAAAAAAAGGGCCTG	GGA-------------------------------GAG
BA	GGGATGCGTAAGCAGGCTCGACTGTTAAGGAGAGGGGCAAATAAGTAAAAAAA-----CTG	GGA-------------------------------GAG
CA	GGG--------------------------------GGCAAATAAGTAAAAAAAAGGGCCTG	GGA-------------------------------GAG
AB	GGGATGCGTAAGCAGGCTCGACTGTTAAGGAGAGGGGCAAATAAGTAAAAAAAAGGGCCTG	GGACCCTTTAGGGGGCTCGACCATAGGGAGAGGAGAG

^1^Polymorphisms detected in the intron 1 of the mitochondrial gene *nad7 *and in the intron B/C of the mitochondrial gene *nad1 *in Scots pine, and nomenclature used for multi-locus haplotypes (mitotypes).

**Figure 1:**
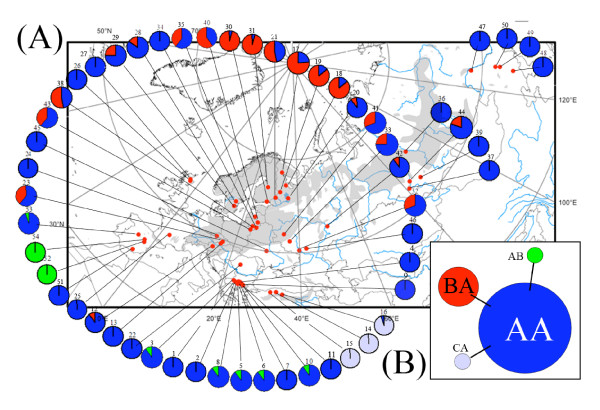
**Geographic distribution of multi-locus mtDNA haplotypes (mitotypes) in Scots pine natural populations from Europe and Asia**. (A) Gray color represents the natural range of Scots pine. The colours corresponding to the mitotypes are defined in plate B. Note a large mixed zone involving the ancestral lineages AA and BA over northeastern Europe. (B) Haplotype network of the four mitotypes identified in this study. Each link represents one indel. The size of circles is proportional to the relative frequency of mitotypes (for exact frequencies, see Table 3).

For Scots pine, the nomenclature used for mitochondrial multi-locus haplotypes (mitotypes) followed a two-letter code after the haplotypes observed for *nad7 *intron 1 (A, B, C) and *nad1 *intron B/C (A, B), respectively (Table 2). Among the four observed mitotypes, the most abundant one was AA (72.3 %), followed by BA (17.3 %), CA (5.8 %), and AB (4.6 %) (Table 3). The minimum spanning tree indicates that the mitotypes were more or less arranged as a star phylogeny with little sequence variation among them and no recombination (Figure 1B). The observed number of mitotypes per population (*nh*) was 1 or 2 without exception, with an average of 1.50 (Table 3). The mitotype diversity index per population (*H*) was low to moderate (average of 0.141) and ranged from 0 to 0.500 (Table 3). Population differentiation was high among the 54 populations, with *G*_ST _and *N*_ST _values of 0.657 and 0.685, respectively (Table 4). When excluding the six more diverse populations from a presumed zone of contact in central and northeastern Europe between two distinct mtDNA lineages (see below), the *G*_ST _and *N*_ST _values increased to 0.746 and 0.767, respectively, among the remaining populations. However, the presence of a formal phylogeographic structure was not supported, as *N*_ST _was not significantly higher than *G*_ST_. Lack of test power is likely the reason, given the small genetic divergence observed among the four mtDNA variants detected.

**Table 3:** Multi-locus haplotype frequencies and genetic diversity estimates in 54 natural populations of Scots pine.

Population		State^1^	Latitude (N)	Longitude^2 ^(E/w)	Altitude (m)	Sample size	Mitotype counts^3^				*nh*^4^	*H*^5^
								
Nb	Name						AA	AB	BA	CA		
1	Bansko	BG	41°48'	23°30'	1400	16	16	-	-	-	1	0
2	Borovo	BG	41°30'	23°55'	1500	18	18	-	-	-	1	0
3	Chehliovo	BG	41°53'	23°55'	1600	20	19	-	-	-	2	0.095
4	Chiroka Laka	BG	41°41'	24°35'	n.a.	20	20	-	-	-	1	0
5	Dospat	BG	41°40'	24°30'	1550	10	9	1	-	-	2	0.180
6	Laki	BG	41°51'	24°50'	1400	20	18	2	-	-	2	0.180
7	Nevestino	BG	42°06'	22°42'	1850	20	20	-	-	-	1	0
8	Pechtera	BG	42°03'	24°22'	1400	20	19	1	-	-	2	0.095
9	Simitli	BG	41°53'	23°10'	n.a.	20	20	-	-	-	1	0
10	Smolian	BG	41°30'	25°20'	1400	20	18	2	-	-	2	0.180
11	Velingrad	BG	42°05'	24°00'	n.a.	20	20	-	-	-	1	0
12	Grosser Priel	AT	47°42'	14°17'	620	20	18	-	2	-	2	0.180
13	Merkenstein	AT	48°59'	16°08'	550	19	19	.-	-	-	1	0
14	Ilgaz Gakdake	TR	41°02'	33°47'	1500	18	-	-	-	18	1	0
15	Eskipazan	TR	40°53'	32°20'	1550	20	-	-	-	20	1	0
16	Ulupihar	TR	40°53'	35°20'	1450	20	1	-	-	19	2	0.095
17	Zelenoborsk	RU	67°10'	32°21'	n.a.	17	4	-	13	-	2	0.360
18	Sosnovec	RU	64°30'	34°45'	n.a.	20	3	-	17	-	2	0.255
19	Sucoozero	RU	63°00'	32°21'	n.a.	20	3	-	17	-	2	0.255
20	Shala	RU	61°47'	36°00'	n.a.	19	17	-	2	-	2	0.188
21	Sortovala	RU	61°42'	30°41'	n.a.	15	7	-	8	-	2	0.498
22	Cugir seed orchard	RO	45°52'	23°23'	230	14	14	-	-	-	1	0
23	Kurim Tisnov	CZ	49°30°'	16°30'	400	12	7	-	5	-	2	0.486
24	Luzna Olesna	CZ	50°10'	13°70'	390	11	11	-	-	-	1	0
25	Murat	FR	45°06'	2°15'	n.a.	9	9	-	-	-	1	0
26	Balnagowan Wood	UK	57°16'	3°09'	240	14	14	-	-	-	1	0
27	Morayshire	UK	57°33'	3°29'	n.a.	20	20	-	-	-	1	0
28	Hallestad District	SE	58°46'	15°35'	85	13	11	-	2	-	2	0.260
29	Rumsulla District	SE	57°41'	15°35'	150	20	15	-	5		2	0.375
30	Kiuruvesi	FI	63°40'	26°40'	n.a.	19	1	-	18	-	2	0.100
31	Vehkalahti	FI	60°35'	27°20'	n.a.	20	1	-	19	-	2	0.095
32	Voronezh	RU	50°30'	40°00'	n.a.	19	13	-	6	-	2	0.432
33	Orlovsk	RU	52°30'	37°00'	n.a.	20	15	-	5	-	2	0.375
34	Kaunas	LT	54°45'	24°05'	100	20	20	-	-	-	1	0
35	Riga	LV	56°53'	24°08'	n.a.	20	11	-	9	-	2	0.495
36	Krasnoyarsk	RU	60°00'	90°00'	n.a.	20	20	-	-	-	1	0
37	Spirinsk	RU	54°00'	81°00'	n.a.	20	20	-	-	-	1	0
38	Vilnius	LT	54°38'	25°28'	100	20	10	-	10	-	2	0.500
39	Krasnoe	RU	54°00'	86°20'	n.a.	20	20	-	-	-	1	0
40	Rokiskis	LT	55°48'	25°33'	120	20	8	-	12	-	2	0.480
41	Kaluzhkaya Ob.	RU	54°00'	35°00'	n.a.	20	12	-	8	-	2	0.480
42	Tatarskaya Ob.	RU	55°00'	50°00'	n.a.	20	19	-	1	-	2	0.095
43	Dainava	LT	53°55'	23°40'	80	20	12	-	8	-	2	0.480
44	Novosibirsk	RU	55°05'	82°45'	200	20	16	-	4	-	2	0.320
45	Kievskaya Ob.	UA	50°00'	30°00'	n.a.	20	20	-	-	-	1	0
46	Groenendaal	BY	50°50'	42°10'	n.a.	18	18	-	-	-	1	0
47	Baiyinna-Heilongjiang	CN	52°21'	125°40'	n.a.	17	17	-	-	-	1	0
48	Jilin Prov.	CN	43°00'	126°00'	n.a.	17	17	-	-	-	1	0
49	Sung-Hua-Chiang	CN	46°00'	127°00'	700	17	17	-	-	-	1	0
50	Heilongjiang Prov.	CN	47°00'	127°00'	n.a.	20	20	-	-	-	1	0
51	Sierras Penibeticas	ES	37°20'	2°50'w	2000	18	18	-	-	-	1	0
52	Montes Universales	ES	40°20'	1°50'w	1700	19	-	19	-	-	1	0
53	Guadarrama	ES	40°45'	4°05'w	1900	19	18	1	-	-	2	0.100
54	Alto Tago	ES	40°44'	2°10'w	1500	18	-	18	-	-	1	0
**Total**						986	713	45	171	57	-	-
**Average**						18.3	-	-	-	-	1.50	0.141

^1^AT, Austria; BG, Bulgaria; BY, Belarus; CN, China; CZ, Czech Republic; ES, Spain; FI, Finland; FR, France; LT, Lithuania; LV, Latvia; RO, Romania; RU, Russian Federation; SE, Sweden; TR, Turkey; UA, Ukraine; UK, United Kingdom.

^2^The "w" indicates the longitude west of Greenwich meridian.

^3^The first letter of multi-locus haplotype (mitotype) refers to the haplotype observed at locus *nad7 *intron1 and the second letter refers to the haplotype observed at locus *nad1 *intron B/C following nomenclature in Table 2.

^4^Number of distinct mitotypes per population.

^5^Mitotype diversity.

**Table 4:** Genetic diversity parameters and population differentiation estimates^1^.

Group	Populations	Region (Country)	*N*^2^	*hh*^3^	*A*^4^	*H*^5^	*G*_ST_^6^	*N*_ST_^7^	*F*_CT_^8^
I	1, 2, 3, 4, 5, 6, 7, 8, 9, 10, 11, 12, 13, 20, 21, 22, 23, 24, 25, 26, 27, 28, 29, 32, 33, 34, 35, 36, 37, 38, 39, 40, 41, 42, 43, 44, 45, 46, 47, 48, 49, 50, 51, 53	Most populations from Europe and Asia	795	3	1.48	0.147	0.262	0.270	0.785
II	17, 18, 19, 30, 31	Karelia and NE Scandinavia (Russia/Finland)	96	2	2.0	0.213	0.001	0.001	
III	14, 15, 16	Middle East (Turkey)	58	2	1.33	0.032	0	0	
IV	52, 54	Iberian Peninsula (Spain)	37	1	1.0	0	n.a.	n.a.	
**Total**			986	4	1.50	0.141	0.657	0.685	

^1^Estimates for groups of populations of Scots pine delineated by SAMOVA and maximizing geographic structure.

^2^Sample size.

^3^Number of distinct multi-locus haplotypes (mitotypes) observed in each group.

^4^Average number of mitotypes per population in each group.

^5^Average mitotype diversity per population in each group.

^6^Differentiation among populations in each group.

^7^Fixation index using PERMUT software [88].

^8^Differentiation among the four groups.

### Geographic structure

The geographic distribution of mitotypes appeared to be essentially non-random, with clusters of mitotypes geographically quite well delineated (Figure 1A, see also SAMOVA test below). The Mediterranean region showed the highest diversity with three of the four mitotypes revealed in the study. The cosmopolitan haplotype AA was largely distributed and found in most of the populations sampled. Geographic structuring was evident for other mitotypes. Mitotype CA was present only in Asia Minor, in the Pontide Mountains of Turkey (populations #14 to #16). Mitotype AB was found fixed in some high mountain populations of the Iberian Peninsula (Iberian cordillera – Zaragoza/Valencia provinces) (#52 and #54) and was observed as a rare variant in some populations of the Balkans (#3, #5, #6, #8 and #10). The distribution of mitotype BA was quite unexpected: it was largely distributed in the lowland region of middle to northern latitudes in eastern Europe. It was prevalent in the Baltic region and quite frequent in Russia, west of the Ural Mountains. But it was also detected in Siberia (population #44). A large zone composed of populations with variable mixed genetic background (mitotypes AA and BA) was observed in central and northeastern Europe. It includes most if not all populations bearing mitotype BA. The zone extends from the southeast of Russia to the Baltic States (see populations #32, #33, #35, #38, #40, #41, and #43). The within-population mitotype diversity of these populations (*H *= 0.463) was much higher than elsewhere.

SAMOVA was conducted repeatedly by increasing the value of the number of population groups (*K*) until the *F*_CT _value reached a maximum of 0.785, which was obtained for *K *= 4 (Table 4). For *K *= 3, a maximum *F*_CT _value of 0.720 was obtained. The four groups confirmed trends from visual inspection and corresponded to: (I) the majority of populations from Europe and Asia, (II) northeastern Europe, (III) Asia Minor and (IV) the high mountains of the Iberian Peninsula. Group I was the most widespread geographically, including 44 populations (Table 4). It contained mostly the cosmopolitan mitotype AA and, at a lower frequency, mitotype BA and AB. Differentiation among the populations of the group was moderate. Population differentiation within each of the three other groups (II, III, and IV) was smaller. Group II from northeastern Europe was largely dominated by mitotype BA. The two remaining groups III and IV were highly homogenous. Group III regrouped populations from Turkey fixed or nearly fixed for mitotype CA, and group IV regrouped populations from the Iberian Peninsula fixed for mitotype AB.

## Discussion

### mtDNA diversity and population differentiation

DNA sequence variation is low in the mtDNA of conifers [e.g. [47,50]]. Accordingly, little intraspecific variation has been found in the mtDNA of *P. sylvestris *in western Europe, including four RFLP variants for the *cox1 *region [5] and three size variants at the locus *nad1 *intron B/C, of which two were found endemic to the Iberian and Italian Peninsulas [6,30]. The three new size variants reported herein for the locus *nad7 *intron 1 indicate new region-specific variants for northeastern Europe and Asia Minor. No other length polymorphisms or substitutions were detected in Scots pine in spite of the screening of 15 distinct mtDNA regions. Highly informative minisatellite-like motifs such as those rarely detected in some spruce [52,53] and pine taxa [20] could not be detected in Scots pine.

The overall level of population differentiation estimated in this study from mitotype variation at the *nad1 *intron B/C and *nad7 *intron 1 loci was rather high and similar to that reported in earlier studies restricted to Scots pine populations from western Europe based on RFLPs of the *cox1 *region (*G*_ST _= 0.83; [5]) or indels of the *nad1 *intron B/C (*G*_ST _= 0.60; [6]). Such estimates are in line with averages reported for maternally inherited markers for a number of conifer and angiosperm taxa [54]. The amount of population differentiation determined by nuclear markers such as isozymes was generally much smaller in Scots pine with *G*_ST _values ranging between 0.025 and 0.076 [43,55-61]]. However, these studies were carried out mostly at the regional level. Among-population differentiation was also much higher for mtDNA markers than for paternally inherited cpDNA markers in a Scots pine study covering much of the species range (*G*_ST _= 0.11; [62]), as usually observed in other conifers [e.g. [54,63]].

### Geographic structure

The present study confirms the existence of a genetically distinct set of populations in the high mountains of the Iberian Peninsula [5,6] while variants discovered in the intron 1 of *nad7 *signal distinctive evolution in Asia Minor and northeastern Europe. Haplotype B at locus *nad1 *intron B/C was observed mostly in the Iberian Peninsula in some high elevation populations of the Zaragoza/Valencia regions where it was fixed or nearly fixed. It was observed only sparingly west of Catalonia. Such a distribution indicates that these Catalan populations have been isolated during glaciations and have not contributed to the recolonization of Europe [[5,6] and [30]]. A distinct dynamics of Catalan populations from the rest of the Iberian Peninsula has also been reported for other species like Aleppo pine [64], Maritime pine [9,65], and white oaks [8]. In the present study, haplotype B of *nad1 *intron B/C has also been discovered, albeit at low frequency, in five other populations located in Bulgaria, in the Balkan Peninsula, thus far away from the center of prevalence for this haplotype in the Iberian Peninsula. Long-distance seed dispersal could be invoked but is unlikely over such long distances, given the heterogenous topography and relative isolation of Iberia. The probability of such long-distance movements would be higher in the northern and more eastern parts of Scots pine natural range, given the lack of major physical barriers. Another perhaps more plausible explanation for the rare presence of haplotype B of *nad1 *intron B/C in some Balkan populations could be that it is ancient and was present in more than one southern ice refugia. Its high or low frequency, depending on the region considered, could be attributable to stochastic variation, especially if southern refugia populations suffered from severe bottlenecks. The weak occurrence of this haplotype in the Balkans might also result from human activity, as previously suggested for the rare presence of this variant in Poland [6]. Whereas the hypothesis of seed source transfer cannot be totally ruled out, it is difficult to sustain because of (i) the lack of hard evidence for such seed source transfer between the Balkans and the Iberian Peninsula in the past; (ii) natural stands in which seeds were collected existed long before forest management activities began in Europe; and (iii) the relative conservatism of forest seed transfer guidelines in the Balkan region [66]. A third explanation could relate to ancient mtDNA capture from hybridizing species in the region [67,68]. Accordingly, phylogeographical studies of other pine species in the region using the same gene locus might help clarify the issue.

The detection and high prevalence of haplotype C of *nad7 *intron 1 in Asia Minor populations only is a strong signal for the presence of a genetically distinct glacial population. This relictual population would have experienced severe bottlenecks, because populations were all fixed for this haplotype except for one tree harbouring the cosmopolitan haplotype A. After glaciation, it would have remained isolated, eastward by the Caucasus Mountains, northward by the Black Sea, and westward by the Bosphorus passage. The cold and arid climate in this region during the Holocene might have also impeded the expansion of this population [69], but not its survival as Scots pine can survive under very harsh conditions [70].

The high prevalence of haplotype B of *nad7 *intron 1 in northeastern Europe suggests the presence of a genetically distinct glacial population at more northern latitudes than previously thought, an idea that had already been suggested for Scots pine [5]. A recent mutation during the Holocene giving rise to haplotype B of *nad7 *intron 1 is unlikely, given the widespread distribution of this haplotype. As well, homoplasy is unlikely, given the low rate of variation of angiosperm and conifer mitochondrial introns [47,49]. A large number of populations had mixed composition of both haplotypes A and B of *nad7 *intron 1 in central and northeastern Europe. Their diversity index was highest among all populations surveyed, which could result for the meeting of two genetically distinct glacial lineages (A and B), or indicates that the glacial population harboring haplotype B had mixed genetic composition. The uneven distribution of haplotype B of *nad7 *intron 1 among these populations, with a higher prevalence of haplotype B as one moves northward, could result from successive founder events during colonization [71], as recently proposed for European hedgehogs [72]. This is likely, given than that migration from more southern latitudes was necessary to colonize these previously glaciated areas.

### Implications for glacial and postglacial history of European vegetation

There is some evidence from fossil and palynological data that cold adapted tree species occupied mid-altitude sites in the mountains of southern Europe during the last glacial stage [13]. Moreover, the southern peninsulas of Iberia, Italy, the Balkans, and Greece, which are separated from the rest of Europe by major mountain ranges such as the Pyrenees, Alps and Caucasus, were identified as major glacial refugia for many species [[14,30] and [65]]. Our genetic data indicates that a genetically distinct glacial refuge existed in Asia Minor as well. Tree pollen, including that of the genus *Pinus*, was recorded in the Balkans about 14 000 years BP [13] while it was barely present as late as 10 500 years BP in southwestern Turkey where the climate was cold and dry with steppe vegetation [69]. Pine pollen attained its maximum only in early Holocene in western Anatolia and in mid-Holocene in more eastern areas, likely because of similar climatic limitations [73,74]. Thus, it is apparent that tree populations would have been scarce in the region at LGM, 21 Kyr ago. The refuge is also unlikely to have contributed much to the postglacial colonization of Europe, given the genetic discontinuity detected in Scots pine. The peculiar location of Turkey between the Black and Mediterranean seas would have promoted isolation from more northern areas. It is likely that other elements of the steppe vegetation in the region also represent relicts from the pre-Holocene era and suffered similar effects during glacial periods, as shown recently for field mouse [75]. As such, it is expected that they would show a distinct genetic background, as that detected in Scots pine.

The exact location and size of the Scots pine glacial population giving rise to the large spread of haplotype B of *nad7 *intron 1 in eastern or northeastern Europe remain unknown. Based on fossil data, Scots pine glacial populations existed in the Balkans and possibly even in central Europe [Litynska-Zajac M. cited by Stewart and Lister [22,23]]. The large spread of haplotype A of *nad7 *intron 1 throughout Europe is likely the consequence of an expansion from glacial populations from that region [30]. Such spread northwestwards towards Germany and France and northeastwards throughout Eastern Europe and Siberia correlates geographic dispersion patterns and colonization routes inferred for a grasshopper [76], for European beech [77], for the oaks [4], and for Norway spruce [52,78]. However, some of these colonization routes are still open to alternative interpretation. Furthermore, such observations do not correlate well with the exceptionally northern location of haplotype B of *nad7 *intron 1.

The peculiar northern distribution of haplotype B of *nad7 *intron 1 indicates that Scots pine has likely survived in a refuge distinct from those usually recognized in the southern peninsulas of Europe, perhaps under the form of scattered glacial populations spread over sizeable latitudinal areas reaching European mid-latitudes not far from the ice front. Scots pine is known to survive and grow reasonably well upon permafrost [72]. Evidence is also accumulating regarding the existence of glacial populations located at more northern latitudes [e.g. [25,28] and [29]] and their significant contribution to Holocene colonization [22,25]. In addition, if the mixed zone observed in north-central and northeastern Europe between the cosmopolitan AA mitotype and the northern BA mitotype represents truly a zone of secondary contact, the northern location and shape of this zone would not correspond well to the meeting of two ancestral lineages both located in or near southern European peninsulas. While the existence of such zones with typically higher genetic diversity has been reported for other tree species in Europe [[3,4] and [16]] as well as in North America [19,20] where ancestral lineages from southern latitudes would converge at more northern latitudes, this possible zone of contact would more likely correspond to the junction between a lineage moving northwards from the Balkans or from even more east (the AA lineage), and a lineage of more northern or northeastern origin (the BA lineage). In one rare occurrence of similar observation, Taberlet *et al *[3] recognized a contact zone of migration fronts as far north as in Scandinavia for *Picea abies*. They proposed that the area was presumably colonized from the south and from the northeast by populations originating from different refugia.

While the current areas of maximum abundance of haplotype B of *nad7 *intron1, Finland and northwestern Russia, were completely glaciated at LGM [79], it is likely that trees colonizing the region would have migrated northward from a region anywhere west of the Ural Mountains. There are indications from general circulation models that the climate during the last glacial maximum was warm enough to allow Scots pine to survive well as far north as above 50°N in isolated regions of northern Ukraine and southwestern Russia, for instance [30]. A location more south is also possible if founder events after long-distance dispersal are hypothetized, followed by drift and lineage sorting to account for the high abundance of haplotype B of *nad7 *intron 1 as one moves into Finland and northwestern Russia. However, this hypothesis becomes unlikely for long-distance dispersal from refuges located in the southern European peninsulas, given the absence of haplotype B of *nad7 *intron 1 in southern populations of Scots pine.

More mtDNA polymorphisms will need to be discovered in order to distinguish Scots pine disjunct populations from eastern Europe and Asia and investigate if significant glacial vicariance factors existed in this vast region. Siberian populations where the cosmopolitan mitotype AA was dominant might have been established from a glacial population located in southwestern Siberia, in the upper Irtysh River Valley [80-82], or from a more southern location. As for Scots pine populations located in the far-east in China where the cosmopolitan mitotype AA was found fixed, it is not clear if it is derived from a more western refuge located in southwestern Siberia (see above) or if a refuge existed in northeastern China. Palynological evidence suggests that pine was present at several locations in the region during the last glaciation, but it is unclear if the fossil pollen was representative of Scots pine as many pine species are endemic to the region [82].

## Conclusion

The present study suggests a more complex biogeographical history than previously thought for Scots pine and presumably, for its associated plant congeners. The study is congruent with others [[3,15,17] and [21]] about the asymetry of glacial refugia in contributing to the postglacial recolonization of Eurasia. This trend is best exemplified by the prevalent *nad7 *intron 1 haplotype found specific to the relictual population from Turkey and from nowhere else in Eurasia. It indicates that Asia Minor is likely to represent a prime area of endemism supporting genetically distinct lineages unlikely to be found in the rest of Eurasia. Such a trend highlights the geography of the region as an important vicariance factor limiting migration and gene flow. Similarly as for other areas of endemism in Europe (e.g. Iberian and Italian Peninsulas), seed source movements from the rest of Eurasia to Asia Minor should be restricted. Reserves could be considered to ensure *in situ *preservation of relictual genetic diversity and structure, which need to be further documented at the level of the chloroplast and nuclear genomes as well as for other plant and animal species.

The discovery of a genetically distinct North-European lineage suggests that some glacial populations may have survived isolated from refugia in South European peninsulas at more mid- northern latitudes. Given the colonization time lag for southern populations to reach northern latitudes due to distance, topographic barriers [2,83] and competition from a possible network of resident populations sparingly distributed, such genetically distinct glacial populations have apparently played a major role in the postglacial colonization of parts of northern Europe. A likely consequence of such glacial vicariance is that extant northern populations of nordic plant species might not be as homogenous as assumed by simply considering them as offsets of glacial populations located at southern latitudes. This observation implies that they might have evolved distinctive genetic adaptations during glacial vicariance, worth evaluating and considering in conservation programs [84,85]. As well, the extensive mixing of two ancestral lineages in central and northeastern Europe provides the opportunity to perhaps identify and preserve unique adaptive diversity.

## Methods

### Population sampling and DNA isolation

Fifty-four natural populations were sampled and their location was chosen in order to represent most of the natural range of the species in Europe and Asia. Twenty populations were from the eastern part of the Ural Mountains and Asia (Russia and China) as well as from Asia Minor (Turkey), and the remaining 34 populations from 13 European countries (Figure 1 and Table 3). Each seed lot represented a bulk of seeds from a minimum of 50 individuals with a minimum distance of 100 m between them. Seed samples were collected from each population and stored in darkness at 4°C until they were processed.

Seeds from each population were soaked on moistened filter paper in Petri dishes at 26°C under a photoperiod of 14 hours for 2 days until DNA extraction. DNA was extracted from the haploid megagametophyte for each of 9 to 20 seeds per population using a genomic DNA mini preparation kit (Sigma-Aldrich), for a total of 986 DNA samples and an average of 18.3 samples per population (Table 3). A screening for mtDNA polymorphism was conducted by assembling an exploratory panel of 26 out of the 986 DNA samples, each representative of a different population and ensuring that geographically widespread populations were represented.

### Screening for mtDNA polymorphism and sequencing

A total of 15 mtDNA regions were screened for polymorphisms using various techniques (Table 1). Polymorphism was detected for only two regions, *nad1 *intron B/C (2 haplotypes), and *nad7 *intron 1 (3 haplotypes). DNA samples bearing different haplotypes for each of the variable mitochondrial regions were selected (3 DNA samples per locus and per haplotype from different populations) and sequenced in order to determine the exact nature of every fragment length polymorphism and to detect the presence of potential fragment length homoplasies. Direct sequencing of the two DNA strands was carried out on an automated *3730XL DNA *analyser (Applied Biosystems) with the dideoxynucleotide chain termination procedure using the appropriate amplification primers and Big Dye Terminator kit-V.3.1 (Applied Biosystems). The complete sequences for *nad7 *intron 1 haplotypes have been deposited in GenBank (GenBank: DQ665913 to DQ665915). The complete sequence for *nad1 *intron B/C was already available in GenBank [6]. Multi-locus mtDNA haplotypes (mitotypes) were then defined by considering single-locus mtDNA genotypes simultaneously and their relationships were determined by statistical parsimony analysis of the aligned sequences using the program TCS [86].

### mtDNA population survey and numerical analyses

Single-locus haplotypes were determined for each of 986 individuals with the internal primers pair *nad1*H and *nad1*I for *nad1 *intron B/C, as described by Soranzo *et al *[6], and the primers *nad7 *intron1 forward and *nad7 *intron1 reverse for *nad7 *intron 1, as described by Jaramillo-Correa *et al *[19]. Primers were synthetized by Invitrogen. DNA was amplified in a PTC225 thermal cycler (MJ Research) using Platinum Taq DNA Polymerase (Invitrogen) in a 12 μl reaction volume, following protocols published by Soranzo *et al *[6] for *nad1 *intron B/C and Jaramillo-Correa *et al *[19] for *nad7 *intron 1. PCR products of *nad7 *intron 1 were digested with the restriction enzyme *Sau*3A I (Promega) and then separated through 8 % non denaturating polyacrylamide gels (in TBE) in order to detect putative cleaved amplified polymorphic sites (CAPS). PCR products of *nad1 *intron B/C were electrophoresed through 2 % agarose gel.

The number of observed mitotypes per population (*nh*) and the total mtDNA diversity per population (*H*; equivalent to the expected heterozygosity, *H*_E_, for diploid data; [87]) were estimated. Population differentiation was estimated using two fixation indices, *G*_ST _and *N*_ST_, using PERMUT [88]. The first statistics is calculated based solely on mitotype frequencies, whereas the second one takes into account the genetic relatedness among mitotypes. Thus, if the estimated *N*_ST _value is higher than the *G*_ST _value, it indicates that closely related mitotypes tend to cluster in the same area, supporting the presence of a formal phylogeographic structure.

Geographic population structure was assessed using a spatial analysis of molecular variance SAMOVA 1.0 [89,90]. A matrix of pairwise distances between populations was first constructed using mitotype frequencies, as well as a matrix of pairwise geographic distances between populations. The method implemented in SAMOVA employs a simulated annealing procedure and uses allele frequency data along with geographic coordinates of the sampled populations to identify population groups maximizing genetic differentiation. We determined the most likely number of *K *groups by repeatedly running SAMOVA with variable numbers of groups and by choosing the number resulting in a maximum *F*_CT _value [90]. The configuration with the largest *F*_CT _value among the 100 tested was retained as the best grouping of populations.

## Authors' contributions

KN was responsible for study design and sampling collection, preparing DNA samples and participated in data analysis and manuscript writing. SS was responsible for PCR assays and DNA sequencing, and participated in data analysis and manuscript writing. JBe was responsible for marker discovery, participated in data analysis, manuscript writing and co-funding of the study. FT contributed to manuscrit review and to co-funding of the study. JBo participated in scaling PCR assays, data analysis, manuscript writing, and co-funding of the study. All authors read and approved the final manuscript.
